# Force versus Response: Methods for Activating and Characterizing Mechanosensitive Ion Channels and GPCRs

**DOI:** 10.1002/adhm.202402167

**Published:** 2024-10-14

**Authors:** Renate M. A. Roeterink, Xevi Casadevall i Solvas, David J. Collins, Daniel J. Scott

**Affiliations:** ^1^ Department of Biomedical Engineering The University of Melbourne VIC Parkville Victoria 3010 Australia; ^2^ Department of Biosystems – MeBioS KU Leuven Willem de Croylaan 42 Leuven 3001 Belgium; ^3^ The Florey Institute of Neuroscience and Mental Health The University of Melbourne Parkville VIC 3052 Australia; ^4^ The Graeme Clark Institute The University of Melbourne Parkville VIC 3010 Australia; ^5^ Department of Biochemistry and Pharmacology The University of Melbourne Parkville VIC 3010 Australia

**Keywords:** G‐Coupled Receptors, ion channels, mechanotransduction, mechanosensitive, patch‐clamp, stimulation

## Abstract

Mechanotransduction is the process whereby cells convert mechanical signals into electrochemical responses, where mechanosensitive proteins mediate this interaction. To characterize these critical proteins, numerous techniques have been developed that apply forces and measure the subsequent cellular responses. While these approaches have given insight into specific aspects of many such proteins, subsequent validation and cross‐comparison between techniques remain difficult given significant variations in reported activation thresholds and responses for the same protein across different studies. Accurately determining mechanosensitivity responses for various proteins, however, is essential for understanding mechanotransduction and potential physiological implications, including therapeutics. This critical review provides an assessment of current and emerging approaches used for mechanosensitive ion channel and G‐Coupled Receptors (GPCRs) stimulation and measurement, with a specific focus on the ability to quantitatively measure mechanosensitive responses.

## Graphical abstract

This review explores techniques used to examine mechanosensitive ion channels and GPCRs, which convert mechanical signals into cellular responses. While these methods have provided valuable insights, discrepancies in reported activation thresholds and responses complicate comparison. The article critically assesses established and emerging approaches for measuring mechanosensitivity, highlighting challenges and opportunities.

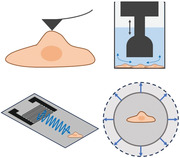

## 1 Introduction

Mechanotransduction is the process whereby biological systems convert mechanical stimuli into electrochemical signals, allowing organisms to sense position, movement, touch, pressure, and pain.^[^ ^1^ ^]^ Cells and tissues are continually exposed to mechanical stimulation and depend on mechanosensitive proteins to convert signals into biological responses.^[^ ^2^, ^3^ ^]^ These mechanosensitive proteins are crucial for the maintenance of many physiological functions, including the vascular system, bone, and skeletal and cardiac muscles.^[^ ^4^, ^5^, ^6^ ^]^ Upon mechanical stimulus, for example, mechanosensitive ion channels switch between closed (inactive) and open (active) pore conformations to allow the passage of ions and other solutes across the cell membrane, whereas mechano‐activated GPCRs couple to intracellular signaling partners to modulate downstream cellular transduction pathways.^[^ ^7^ ^]^ The focus of this review is on mechanosensitive ion channels and GPCRs, where these proteins have been the subject of recent investigation and show promise in generating controlled cellular responses to external mechanical stimulation. However, mechanotransduction extends to a wide range of mechanosensitive proteins.^[^ ^1^ ^]^ These proteins have their own force‐dependent functions and control signaling pathways, thereby regulating transcription programs. For instance, cells sense through adhesion receptors, such as integrins, where integrins are a family of cell surface protein receptors that interact with other cells, extracellular matrix (ECM), and cytoskeleton, transmitting mechanical forces into chemical signals. Integrins further lead to the recruitment of vinculin, zyxin, talin, actin, and other focal adhesion components, which are crucial for intracellular responses to changes in the mechanical environment.^[^ ^8^ ^]^

In recent years, emerging technologies have led to the identification and characterization of mechanosensitive ion channels in a range of organisms, from bacteria to humans. Studies have used molecular, genetic, and electrophysiological techniques to examine the structure and function of mechanosensitive ion channels, using a wide range of experimental methods to gain further understanding of how cells sense mechanical stimulus.^[^ ^9^, ^10^, ^11^, ^12^, ^13^, ^14^ ^]^ Two mechanisms for how mechanosensitive ion channels sense mechanical forces have been suggested, depending on the morphology and function of the specific protein. In the “force‐from‐lipid” mechanism, lipid bilayer tension alone is sufficient to gate an ion channel without the requirements of intra – or/and extracellular structures, as seen in bacterial MscL and MscS.^[^ ^15^ ^]^ The other model, “force‐from‐tether”, hypothesizes that channel opening in response to a mechanical force originating from the interaction between an ion channel with the ECM or cytoskeletal components, or both, where the specific mechanism that predominates may be protein‐dependent.^[^ ^16^, ^17^, ^18^ ^]^

One of the most extensively studied ion channels capable of sensing mechanical stimuli is the bacterial mechanosensitive channel of large conductance (MscL).^[^ ^19^ ^]^ In addition to transporting molecules and ions across the membrane, MscL also functions as a pressure regulator to prevent membrane damage.^[^ ^20^ ^]^ MscL is a valuable tool for studying mechanotransduction as it is capable of functioning in mammalian cells and artificial bilayers.^[^ ^21^ ^]^ However, the half‐maximal pressure required to activate MscL is highly variable, ranging from ‐140 mmHg to ‐35 mmHg.^[^ ^19^, ^22^ ^]^ As a further example, a class of eukaryotic and evolutionarily conserved mechanosensitive proteins is Piezo1 and Piezo2, which are large (>2500 amino acids) pore‐forming ion channels that have gained attention as valuable mechanosensitive candidates since their discovery in 2010.^[^ ^23^ ^]^ The large molecular structure of Piezo channels comprises 38‐transmembrane helices arranged in a three‐bladed, propeller‐like trimer. Highlighting the ubiquity of mechanosensation in cell function, Piezo channels are broadly expressed in mechanosensitive cells found in the lungs, kidneys, and DRG neurons of mammals.^[^ ^24^, ^25^ ^]^ Notably, Piezo1 is involved in multiple physiological processes, including vascular development, vascular physiology, and erythrocyte volume regulation.^[^ ^4^, ^5^, ^26^ ^]^ Piezo1 activation has been demonstrated using a range of stimuli including stretch,^[^ ^27^ ^]^ cellular indentation,^[^ ^23^ ^]^ elastomeric pillar deflection,^[^ ^28^ ^]^ fluid shear stress,^[^ ^29^ ^]^ and ultrasound.^[^ ^30^, ^31^ ^]^ Reconstitution of Piezo1 without endogenous cellular components showed that Piezo1 is inherently mechanosensitive.^[^ ^32^, ^33^ ^]^ Upon activation, Piezo1 transitions from a curved state into a flattened state, which subsequently opens a central pore through the membrane.^[^ ^14^, ^34^ ^]^ However, the origin and magnitude of forces that initiate the transition of this highly sensitive protein remain poorly understood, specifically the lack of quantitative data on the gating thresholds required for Piezo1 activation.

In addition, there is increased evidence that various G protein‐coupled receptors (GPCRs) can sense mechanical force and stimulate intracellular signaling pathways.^[^ ^35^ ^]^ GPCRs are a superfamily of cell surface proteins that serve as sensors for a diverse range of extracellular stimuli. Besides the well‐known activation of GPCRs through diffusible ligand molecules, mechanical stimulation also acts as a mode of activation of some GPCRs. Mechanical force activation was first shown in Angiotensin II receptor type 1 (AGTR1) and has since then led to the discovery of several mechanosensitive GPCRs including bradykinin receptor B2 (BDKRB2), parathyroid hormone type 1 receptor (PTH1R) and GPR68.^[^ ^36^, ^37^, ^38^, ^39^ ^]^ Fluid shear stress and membrane stretch have been identified as two major types of mechanostimulation for GPCRs,^[^ ^35^, ^36^, ^40^, ^41^ ^]^ however, their function as mechanosensitive receptors and the potency at which they are stimulated mechanically are less explored than the prototypical mechanosensitive ion channels such as MscL and Piezo1.^[^ ^42^ ^]^

Recent advances have aided in identifying and characterizing mechanosensitive proteins, though precise quantification of mechanosensitive protein responses remains challenging, especially between different techniques and methods. Accurately deciphering the gating requirements is crucial for understanding mechanotransduction mechanisms and their potential therapeutic applications, however, where force versus response measurements have been investigated, activation thresholds can vary greatly between studies investigating the same mechanosensitive protein. Achieving controlled activation of mechanosensitive membrane proteins through external mechanical forces thus necessitates improved control and standardization on the mechanical loading conditions required for the gating of individual proteins. Furthermore, understanding how cells respond to applied forces is crucial for developing biomaterials that aim to mimic the complexity of tissues, such as nerve, bone, or cardiac biomaterials.^[^ ^43^, ^44^, ^45^ ^]^ The properties of the material, including elasticity, stiffness, topography, and geometry, significantly influence cell behavior. By incorporating specific characteristics into biomaterials, essential mechanical cues can be provided that regulate cellular responses and improve functionality.

While other reviews have examined mechanosensitive protein characteristics and activation mechanisms, here we outline the main mechanical stimulation techniques, discuss their limitations, and examine their ability to obtain accurate quantitative measurements.^[^ ^16^, ^46^, ^47^, ^48^, ^49^, ^50^, ^51^, ^52^ ^]^ Particular attention is given to the quantification of Piezo1 ion channel activation, given the large amount of recent work with this protein and to highlight limitations seen across different measurement methodologies. Further, current techniques and recent advancements in measuring intracellular responses by mechanosensitive ion channels and GPCR activation are examined. Finally, novel and promising approaches are discussed, and their applicability in integrating mechanical stimulation methods and output within physiologically relevant setups.

## 2 Methods for Stimulating Mechanosensitive Ion Channels

**Figure 1:**
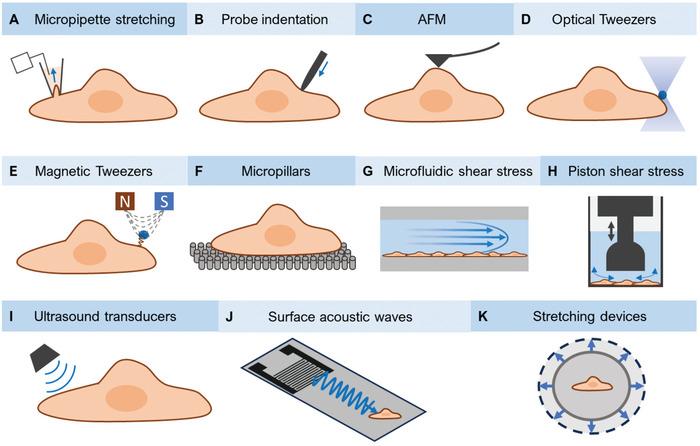
Overview of techniques for mechanosensitive ion channel stimulation. A) Pressure‐clamp technique for stretch activation in cell‐attached mode. B) Indentation of cells by local cellular indentation using a blunt glass pipette. C) An AFM cantilever pushes or pulls on a cell. D) Highly focused laser beam traps and controls microscopic dielectric spherical particles. E) Forces exerted by applying a magnetic field on magnetic particles. F) Cell‐substrate displacement by pillar deflection on a micropillar array. G) Activation through shear forces in a microfluidic unidirectional flow cell. H) Activation through shear forces generated by radial flow in a well format by piston movement. I) Ultrasound stimulation of cells produced by an ultrasound transducer. J) Surface acoustic waves (SAW) on a microfluidic platform stimulation. K) Stretching populations of cells in cell stretching devices.

Various techniques to study mechanosensitive proteins have been established, where these apply physical stimuli to cells and measure physiological responses in cells and cell‐like preparations. Techniques include membrane stretch,^[^ ^53^ ^]^ probe indentation,^[^ ^54^ ^]^ Atomic Force Microscopy (AFM),^[^ ^55^ ^]^ optical tweezers,^[^ ^56^ ^]^ magnetic tweezers,^[^ ^57^ ^]^ micro elastomeric pillars,^[^ ^28^ ^]^ shear stress devices,^[^ ^36^ ^]^ stretch devices,^[^ ^58^ ^]^ and acoustic methods^[^ ^52^ ^]^ (**Figure** **1** and **Table** **1**). In this section we examine the fundamentals of these techniques, examining their applications, advantages, and limitations.

**Table 1:** Summary table of stimulation techniques.

Method	Mechanism	Stimulus range	Application examples	Advantages	Disadvantages	
**Pressure clamp**	Patch‐limited Stretch membrane	0 to ‐120 mmHg	Local stretching of the cell membrane P50 determinations	High precision Control over pressure Real‐time recording Incremental pressure steps within one recording	Low throughput Short term recording Pressure converted to local stresses varies in each cell	[23, 59, 60]
**Probe Indentation**	Indentation	Indentation steps of 0.5‐1µm	Local indentation of the cell membrane	Advantageous for low expression level mechanosensitive proteins	Less specificity Lower throughput due to whole‐cell configuration Induced forces unknown The resulting cell deformation is unknown Only feedback by imaging	[23]
**Atomic Force Microscopy (AFM)**	Indentation Stretch Pulling	10‐40^4^ pN	Individual proteins measurement Cell indentation Single‐molecule manipulation Ligand‐receptor studies High force pulling	Simple force measurements Can be combined with complementary methods: fluorescence microscopy, electrodes, patch clamping	Restricted to cell surface measurements Complex technique Nonspecific Large high‐stiffness probe	[14, 61, 62]
**Optical Tweezers**	Stretching Indentation Pulling	0.1‐100 pN	Single‐molecule manipulation, e.g. DNA, proteins, or enzymes. Detecting changes in force‐responsive domains of mechanosensitive proteins. Target receptors by coating beads with ligand.	Able to apply forces intracellularly Versatile manipulation	Requires accurate force calibration Photodamage Thermal damage	[56, 63]
**Magnetic Tweezers**	Pulling	10^−3^ to 30^4^ pN	Single‐molecule manipulation, e.g. DNA, proteins, or enzymes Fixed force	Able to apply forces intracellularly Stable and constant force over time High throughput	Limited control Limited to magnetically responsive materials Limited to surface‐attached beads	[57]
**Micropillar array**	Pillar displacement	0‐1000 nm pillar deflection ≈<100 nN	Substrate ECM functionalized/influences mechanosensitive proteins Cell behavior, e.g., mobility, direction of migration	Absolute measurements Applies stimuli at the interface between cells and their substrates Can be combined with cell imaging	Small stiffness range Requires microfabrication expertise	[28, 64]
**Shear stress devices: microfluidic flow chamber or pistons**	Shear forces	0‐6400 dyn cm^−2^ Shear stresses are largely dependent on the dimensions of the chamber	Cell migration, orientation, and shape Vascular development investigation Shear‐stress differentiation of cells Drug discovery	Physiologically relevant Can be combined with cell imaging	Achieving uniform shear stress can be challenging Limited throughput Device complexity and fabrication	[4, 36, 65]
**Acoustic methods: Ultrasound transducers or SAW**	Acoustic radiation force Shear forces Cavitation forces Pressure waves	**Transducers**: Several kHz to a few MHz **SAW**: several MHz to GHz	Deep‐brain region stimulation Sonogenetics	In vivo stimulation Can be combined with microfluidic‐chip stimulation	Difficult to translate to physiological environments Difficult to isolate single‐force	[21, 31, 66, 67]
**Stretching devices**	Whole‐cell stretch Substrate‐cell interface stretch	1‐20%	Cyclic strain and stretch Cardiovascular focus Uniaxial, biaxial, equiaxial strain	Can be combined with cell imaging, patch‐clamping, AFM, microfluidic chip	Difficult to translate to physiological environments	[27, 58]

### 2.1 Direct Stimulation of Mechanosensitive Proteins

#### 2.1.1 Pressure Clamp

Pressure clamp is a commonly used technique that applies changes in pressure to an isolated area of the membrane of a cell within the pipette while recording cross‐membrane current (Figure 1A).^[^ ^68^ ^]^ The applied pressure causes changes in membrane curvature, stretching the membrane and changing the membrane tension. Therefore, this technique is beneficial for studying stretch and membrane tension‐activated ion channels. In addition, responses to several pressure differences can be obtained within one reading, where stimulation and recording are performed simultaneously. The P_50_ curve, derived from a pressure response curve fitted with a sigmoidal Boltzmann function, is the pressure of half‐maximal activation and is crucial for assessing mechanosensitive protein sensitivity.^[^ ^69^, ^70^ ^]^ It enables standardized comparisons between experimental conditions and studies by determining the pressure required for half‐maximal activation. P_50_ measurements, commonly conducted in cell‐attached pressure clamp experiments, reveal shifts in protein activation and sensitivity. For instance, comparing Piezo1 to a Piezo1 variant (isoform Piezo1.1), Geng et al. observed a leftward shift in the pressure‐current response curve that indicated Piezo1.1 is approximately twice as sensitive.^[^ ^70^ ^]^ Notably, Piezo1.1 also exhibits distinct Ca^2+^ selectivity and response, highlighting isoform‐specific roles in physiological mechanotransduction processes and diverse biological functions.

**Figure 2:**
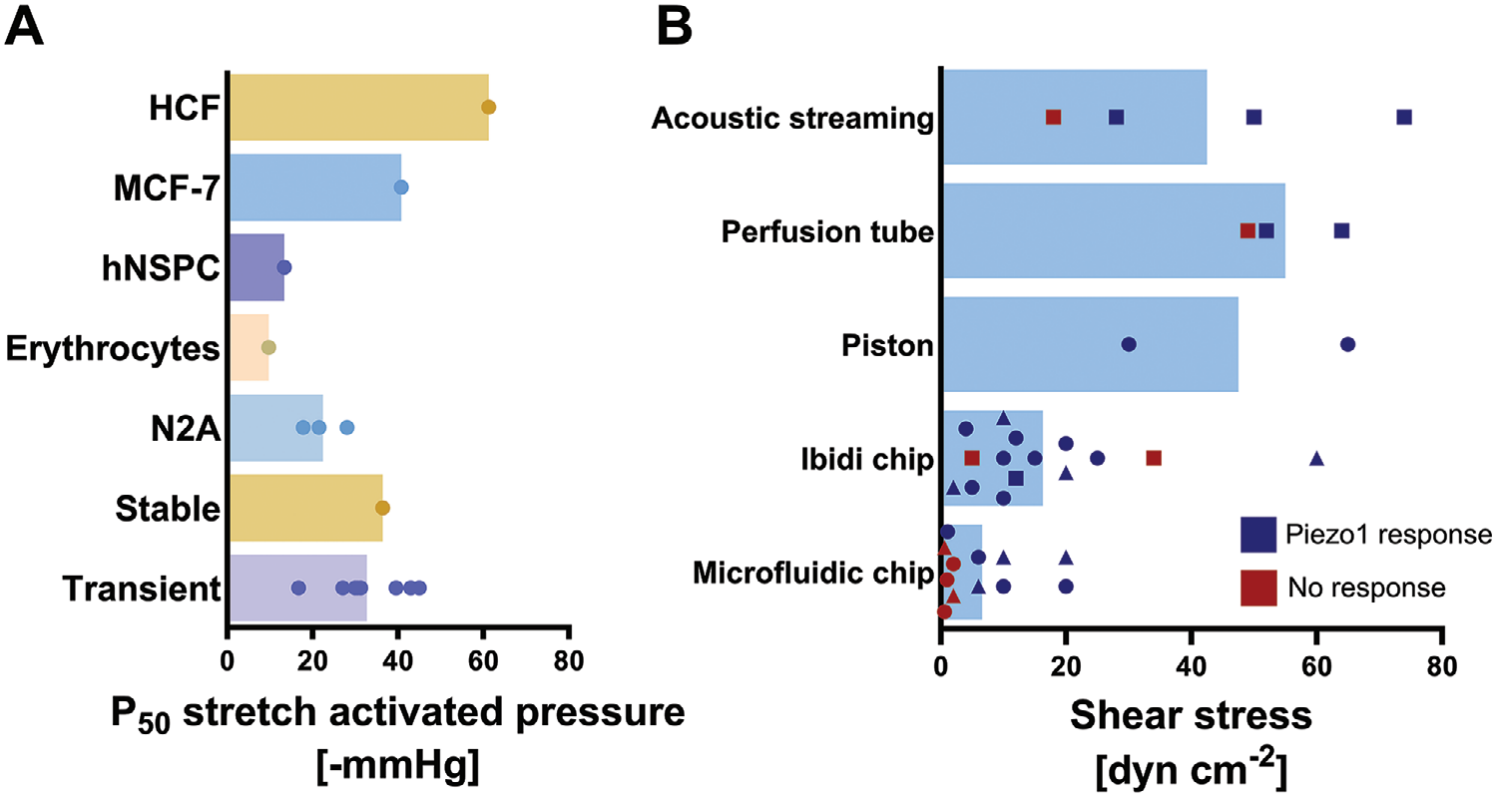
Quantitative characterization of Piezo1 channel activation by cell‐attached pressure clamp and shear force stimulation, derived from reported studies outlined in Table 2 and 3) P_50_ values derived from stretch‐activated pressures for Piezo1 activation measured in different cell types. Stable expression and transient transfected expression of Piezo1 in HEK293T cells. B) Comparison of shear forces used to stimulate Piezo1 expressing cells, that lead to Piezo1 response or no response of Piezo1 by different shear force generating platforms. Data points represent endogenous Piezo‐expressing cells (round), transiently transfected cells (square), and stable Piezo1‐expressing cells (triangle). Dark blue data points indicate Piezo1 activation by the applied shear stress, while red points indicate no observed Piezo1 response upon applied shear stress. All data points are taken from references in Tables 2 and 3.

Highlighting the wide variation in system‐dependent activation of Piezo1, **Figure** **2** examines the range in observed Piezo1 responses as a function of cell type and activation setup, even when employing similar mechanisms. Examining Piezo1 activation thresholds in different cell types via pressure clamp for instance, unveils a P_50_ range from ‐10 to ‐60 mmHg, demonstrating sensitivity variations influenced by factors like membrane tensions, membrane curvature, endogenous cytoskeleton components, and cell membrane compositions (Figure 2A and **Table** **2**).^[^ ^29^ ^]^ Here different cell types have different native membrane tensions and cellular expression and localization of Piezo1, but also differential expression of endogenous cellular components and membrane compositions. The tension‐sensing capabilities of Piezo1 trimers can further be modulated by the cytoskeleton and extracellular matrix composition, which affects the sensitivity of Piezo1 to mechanical stimuli, where the cytoskeleton provides a mechanoprotective role to the cells, thereby desensitizing Piezo1 activity.^[^ ^71^ ^]^

**Table 2:** Cell‐attached negative pressure gating of Piezo1 measured by patch‐clamp recording.

Channel	Cell type	P_50_ [‐]	Holding Potential	Mechanical stimulus [mmHg]	Pipette size [mm] and resistance giga ohm seal [MΩ]	Read‐out	Validation method	Reference
mPiezo1 (transfected)	Neuro2A	21.5 mmHg	−80 mV	0 to ‐80 (Δ‐5)	1.5 OD, 0.85 ID, 3–6.5 MΩ	Current	Neuro2A Piezo1‐KO cells	[60]
Piezo1 (transfected)	Neuro2A	28.1 ± 2.8 mmHg	−80 mV	0 to ‐60 (Δ‐10)	2‐3 MΩ	Current	Mock transfected	[23]
Piezo1 (endogenous)	Neuro2A	17.8 ± 2.2 mmHg	+65 mV	0 to ‐120 (Δ‐10)	2‐3 MΩ	Current	Piezo1‐KO‐HEK293T	[59]
Piezo1 (endogenous)	MCF‐7	40.8 ± 1.1 mmHg	−100 mV	0 to 200 mBar	1.6 OD, 1.0 ID, 1–2.5 MΩ	Current	N/A	[73]
Piezo1 (endogenous)	Human Cardiac Fibroblasts (HFC)	61.3 mmHg	+80 mV	0 to ‐120	4‐6 MΩ	Current	Piezo1‐siRNA transfected	[74]
Piezo1 (endogenous)	Human neural stem cells (hNSPCs)	13.4 mmHg	−80 mV	0 to ‐30 (Δ‐3)	0.6‐0.9 MΩ	Current	GsmTx‐4, Piezo1‐siRNA	[75]
Piezo1 (endogenous)	Erythrocytes	9.72 mmHg ± 0.51	N/A	−5 to ‐25 (Patch‐clamp to capture the cell)	1.5 OD, 0.85 ID, 15–20 MΩ	Ca^2+^	Vav1‐P1cKO RBCs	[26]
Piezo1 (transfected)	HEK293T	31.3 mmHg ± 2.9	−120 mV	0 to ‐70 (Δ‐5)	2‐3 MΩ	Current	Piezo1‐KO‐HEK293T	[70]
mPiezo1 (transfected)	HEK293T	30 mmHg	−80 mV	0 to ‐80 (Δ‐10)	2‐3 MΩ	Current	Non‐transfected	[76]
mPiezo1 (transfected)	HEK293T	27.1 ± 3.1 mmHg	−80 mV	0 to ‐100 (Δ ‐10)	N/A	Current	Piezo1‐KO‐HEK293T	[77]
Piezo1 (transfected)	HEK293T	31.2 ± 3.5 mmHg	−80 mV	0 to ‐60 (Δ ‐10)	2‐3 MΩ	Current	Mock transfected	[23]
hsPiezo1‐GFP (stable)	HEK293T	36.4 ± 3 mmHg	+65 mV	0 to ‐120 (Δ‐10)	3 MΩ	Current	Piezo1‐KO‐HEK293T	[59]
hPiezo1 (transfected)	HEK293T	39.6 ± 2.1 mmHg	+65 mV	0 to ‐120 (Δ‐10)	3 MΩ	Current	Piezo1‐KO‐HEK293T	[59]
Piezo1 (transfected)	HEK293T	30.5 ± 1.7 mmHg	−80 mV	0 to ‐100 (Δ‐10)	N/A	Current	Piezo1‐KO‐HEK293T	[78]
hPiezo1 (transfected)	HEK293T	43 ± 0.7 mmHg	−60 mV	−20 to 70 (Δ‐10)	2‐5 MΩ	Current	GSMTx4	[79]
Piezo1 (transfected)	HEK293T	45 ± 3 mmHg	−60 mV	−10 to ‐50 (Δ‐10)	3.5–5.0 MΩ	Current	N/A	[71]
mPiezo‐IRES‐GFP (transfected)	HEK293T	16.7 ± 2.8 mmHg	−80 mV	0 to ‐60 (Δ‐10)	1.5 OD, 0.85 ID, 1.5‐4 MΩ	Current	Empty vector	[80]

Examining the role of activation methods, the giga‐ohm seal between the pipette and the membrane creates high tensional stresses before the stimulus is applied, making it physiologically different from the resting state of a cell.^[^ ^50^, ^72^ ^]^ Therefore, while quantitative data on the required pressures to activate mechanosensitive proteins can be obtained, the exact force stimulus the channel senses (compared to resting) may not be representative of physiological conditions. Furthermore, it should be considered that the composition of the patch is not identical to the rest of the cell, as patch formation requires redistribution of components, altering the mechanical properties of the patch compared to the resting state of the cell, which is likely reflected in the high variability seen in P_50_ determinations.^[^ ^72^ ^]^ Consequently, data obtained may not be relevant to the same channels in vivo. Moreover, compared to other methodologies this approach is intrinsically low‐throughput, restricted to single‐cell quantification at a given time with activation of a limited number of channels across the “patched” portion of the cell membrane.

#### 2.1.2 Probe Indentation

To obtain channel‐mediated ion flux data from the entire plasma membrane, whole‐cell patch measurement is achieved by increasing the negative pressure and rupturing the membrane patch, allowing electrical access to the whole cell.^[^ ^60^, ^81^ ^]^ Utilizing the whole‐cell patch clamp configuration in combination with local cellular indentation, the actuation, and measurement of numerous channels are achieved (Figure 1B). Here, mechanical stimulation is applied by indentation using a rounded glass probe (2‐5 µm in diameter), causing apical compression of the cell surface and deformation of the membrane. This technique was used in the first identification of Piezo1 and Piezo2 as mechanosensitive proteins.^[^ ^23^ ^]^ Aside from specifically activating mechanosensitive proteins, however, indentation forces also apply load to the cytoskeleton. Consequently, these forces likely propagate throughout the cell‐substrate network, making it unclear which specific molecular mechanisms drive mechanosensitive protein activation.^[^ ^82^ ^]^ Indentation probes further have a low spatial resolution compared to cell dimensions and, in most configurations, lack force‐feedback, thus it can be challenging to accurately quantify the magnitude of the mechanical stimulus that is applied and required to gate single or multiple mechanosensitive proteins. The fact that cells have viscoelastic and plastic properties further means that the local stresses will change over time. The force exerted on mechanosensitive proteins also decreases with the distance from the site of indentation. Moreover, the localization of mechanosensitive proteins is not homogenously distributed across the cell membrane, where different indentation locations can lead to different quantitative outcomes.^[^ ^50^ ^]^

#### 2.1.3 Atomic Force Microscopy

AFM in the context of protein measurements is a powerful and well‐established indentation methodology that applies and measures force in the pico to nano Newton range at nanometre spatial resolution in biological systems at cellular and subcellular levels.^[^ ^61^ ^]^ In AFM, a cantilever with a probe is used to detect forces between the probe and the sample, measuring the mechanical properties of the cell, and thus ideal for mechanotyping purposes (Figure 1C). The cantilever is controlled by a piezoelectric positioner and interacts with a cell by pushing or pulling on the surface, thereby evoking mechanosensitive protein activity.^[^ ^14^ ^]^ For example, High‐speed AFM investigation of Piezo1 reconstituted into lipid membranes revealed that the dome structure of Piezo1 flattens reversibly under applied force to the membrane surface.^[^ ^14^ ^]^ Using precisely controllable subcellular‐scale displacements, Gaub et al. stimulated different regions within cortical neurons and demonstrated that the soma, dendrites, and axon regions respond differentially to mechanical stimulation pressures.^[^ ^62^ ^]^ Additionally, the AFM cantilever can be functionalized, for example with different extracellular matrix proteins or a ligand, to examine specific interactions of the mechanisms involved in mechanosensitive protein response.^[^ ^83^ ^]^ Here an AFM probe with ECM proteins resulted in increased Piezo1 sensitivity to mechanical forces and with channel activation occurring at a significantly lower mechanical pulling force, though it is unclear whether ECM components either act directly or indirectly on Piezo1 through surrounding membrane‐bound proteins.^[^ ^61^ ^]^ AFM probes, however, are generally limited to stimulating components of the cell surface, and unable to explore intracellular dynamics. To overcome this limitation, a modified AFM tip that penetrates the cell membrane allows for intracellular probing of mechanical properties.^[^ ^84^ ^]^ Moreover, to provide depth to AFM recordings, studies have combined force readouts with optical imaging, recordings of electrical activities measured by multielectrode arrays (MEA) and patch‐clamp devices to link the mechanically triggered biological response.^[^ ^54^, ^55^, ^85^, ^86^ ^]^ However, AFM techniques, like patch‐clamp techniques, require time‐consuming sample preparation and data acquisition, and handle only a single cell at a time, which considerably limits throughput.

#### 2.1.4 Optical Tweezers

Optical tweezers, also known as optical traps, use a highly focused laser beam to trap and control microscopic dielectric spherical particles, allowing the measurement of sub‐pN forces with microsecond temporal resolution and generating forces ranging 0.1 to 100 pN (Figure 1D).^[^ ^87^ ^]^ The laser beam creates a gradient force, where particles are attracted to the beam's center where the intensity is highest. This technique has been used on single molecule perturbation, including DNA and RNA, related protein interactions, and can be used to measure mechanical properties of cellular components, such as stiffness and elasticity. Additionally, the particles can be coated with ligands of interest and used to target specific receptors on the cell surface. Further, in contrast to AFM where the cantilever obstructs optical access, optical tweezers can investigate intracellular compartments with minimal mechanical and optical obstruction.^[^ ^56^, ^63^ ^]^ Optical tweezers have been used to investigate membrane deformations and cytoskeletal structures related to the functioning of mechanosensitive ion channels. Highlighting the ability for optical tweezers to generate mechanical forces on subcellular length scales, Shi et al. demonstrated that changes in membrane tension do not propagate over long distances in the plasma membrane, evidencing the ability to generate localized mechanosignalling.^[^ ^88^ ^]^ Interestingly, localized mechanical forces of 5.5 pN applied to the actin cytoskeleton directly activated mechanosensitive channels near the focal adhesions in HUVECs, highlighting the critical role of the cytoskeleton in the activation of these channels.^[^ ^89^ ^]^ However, although optical tweezers gain many advantages from using light, the intense laser illumination required for optical trapping can cause difficulties, including photo and thermal damage to the sample.

#### 2.1.5 Magnetic Tweezers

Magnetic micromanipulation by magnetic tweezers is a versatile technique through which forces are exerted by applying a magnetic field on magnetic particles (Figure 1E).^[^ ^90^ ^]^ In a permanent magnet configuration, the force is controlled by the rotation of the magnets, causing the magnetic particles with attached molecules to be attracted toward the magnetic field. The force on a magnetic particle changes minimally with rotation, with magnetic force gradient length scales arising from typical permanent magnets being far larger than cellular dimensions, making magnetic tweezers ideal for studying targets that require constant force stimulation.^[^ ^91^ ^]^ While such a force‐clamp can also be achieved with AFM and optical tweezers, this typically requires sophisticated active feedback systems.^[^ ^92^ ^]^ Wu et al. applied local forces to distinct domains of Piezo1 through magnetic nanoparticles and identified two mechanically sensitive domains required for activation and inactivation of the ion channel.^[^ ^57^ ^]^ Magnetic tweezer experiments however are limited to certain experimental setups, as they require the attachment of beads to the molecule or structure of interest. This attachment can potentially interfere with the biological function by altering the mechanosensitive ion channel or GPCR conformation or blocking important binding sites. Furthermore, magnetic tweezers are limited by their reliance on image analysis to track the 3D positions of magnetic particles, which constrains time resolution compared to faster techniques like AFM and optical tweezers. Recent advancements in improved hardware and faster image acquisition technologies have enhanced the resolution and speed, allowing monitoring of nanoscale changes in target molecules.^[^ ^93^, ^94^ ^]^

#### 2.1.6 Elastomeric Micropillars

Whereas indentation techniques are limited in throughput where stresses are exerted on both the membrane and the underlying ECM, an alternative method utilizes elastomeric micropillars to monitor mechanotransduction at defined regions at the cell‐substrate interface.^[^ ^28^ ^]^ This technique cultivates adherent cells on elastomeric polymer structures with defined dimensions and mechanical properties, where mechanical stimulation is applied by deflecting a single pillar subjacent to the cell, thereby stimulating a small number of channels surrounding the pillar (Figure 1F). Force measurements on the order of 1 nN can be deduced from micropillar displacements, while the cellular response is recorded by whole‐cell patch clamp electrophysiology or microscopy‐based assays. When compared to cell indentation techniques, a reduced stimulus is required to activate a similar magnitude of mechanosensitive currents in DRG neurons and N2A cells.^[^ ^28^ ^]^ Achieving precise control over the applied force can be challenging, however, as pillars offer a limited force range and do not necessarily demonstrate linear mechanical deformation. Accordingly, elastomeric micropillars have been most useful in understanding how the mechanical properties of the substrate influence mechanosensitive protein activity, rather than serving as a precise and controlled mechanical force stimulation tool.

#### 2.1.7 Shear Stress Devices

Fluid shear stress is a physiological‐relevant mechanical force exerted on cells exposed to fluid flow (e.g., blood), where shear stresses induce lipid bilayer deformation and consequently modulate the membrane tension.^[^ ^29^, ^47^ ^]^ In the case of GPCRs, changes in cell membrane tension affect the conformational dynamics of GPCRs.^[^ ^40^ ^]^ Mechanical shear stresses in vivo have a significant effect on endothelial physiology, hence different blood flow patterns lead to different functions and implications.^[^ ^95^ ^]^ Highlighting the importance of shear stress sensing in blood flow, higher shear stresses (10‐70 dyn cm^−2^) represent an atheroprotective level of shear stress, where shear stress is essential for elongation, intact barrier function, and maintaining vessel health.^[^ ^96^ ^]^ In contrast, low shear stress levels (<4 dyn cm^−2^) in arteries are associated with disturbed flow patterns, which lead to inflammation and are considered atherogenic.^[^ ^97^ ^]^ Under high laminar shear flow, endothelial cells elongate and align in the direction of flow, whereas cells under disturbed flow are stimulated from many different angles and become thus randomly orientated.^[^ ^95^ ^]^ Therefore, when using fluid as a shear stress stimulator in an experimental design set‐up, important factors need to be considered to recapitulate physiologically relevant processes.

A standard approach for investigating the effect of shear stress is by culturing cells in a parallel‐plate flow chamber, where populations of cells are subjected to controlled shear stresses and responses are monitored, for example, by optical techniques or gene expression (Figure 1G).^[^ ^98^, ^99^, ^100^ ^]^ A limitation of these methods is the inflexibility to change design parameters, where it is impossible to analyze the effect of several shear stresses at once in a single setup. As such, a variety of systems have been developed to model fluid flow and shear stress exposure upon cultured cells. Baratchi et al. developed a microfluidic device with varying geometries to create several shear stresses that are visible in one field of view to observe responses across many cells simultaneously.^[^ ^101^ ^]^ This microfluidic platform permitted the examination of shear stresses from 0.6 to 20.5 dyn cm^−2^, thereby examining a range of physiological and pathophysiological shear stress ranges simultaneously. Analysing bovine aorta endothelial cells, they measured a half‐maximal shear stress effect (SS_50_) of 5 dyn cm^−2^, with increasing shear stresses leading to increased cell sensitivity observed by quicker response times. However, many flow chamber set‐ups consist of a fixed assembly of parts that is completed before seeding cells, considerably limiting the flexibility of such assays. Therefore, fluorescence microscopy is usually used to record cellular responses, which itself is time‐consuming and labour‐intensive.

Besides microfluidic approaches, other microscale platforms based on current molecular cell culture tools have been developed to allow for higher throughput mechanical stimulation systems. Fonseca et al. developed a modular 96‐well fluidic platform to perform high throughput screenings under continuous flow‐induced shear stress, though this was limited in shear stresses up to 7 dyn cm^−2^.^[^ ^102^ ^]^ Interestingly, Xu et al. identified G protein‐coupled receptor GPR68 to be responsive to shear stress using a custom high‐throughput 384‐well oscillating pistons device, generating shear stresses up to 167 dyn cm^−2^ (16.7 Pa) (Figure 1H).^[^ ^36^ ^]^ The same study demonstrated that disturbed shear stresses, but not steady laminar flow, could activate Piezo1‐dependent Ca^2+^ transients in HEK293T cells transfected with mouse Piezo1. This screening device allows for quick determination of flow‐sensing proteins, though the shear forces in this acoustically driven setup are difficult to characterize, non‐uniform, and are poorly controlled within wells. Conversely, Segeritz et al. developed a 96‐well plate‐compatible plug‐in device that generates controlled uniform laminar flow within a single well.^[^ ^38^ ^]^ The device creates several shear forces across the radial position, thereby controllably stimulating an expressed protein (GPR68) with a range of shear forces in the same measurement. Through assaying intracellular Ca^2+^ levels with a plate reader across a tuneable range of shear stresses, Segeritz et al. estimated the SS_50_ of GPR68 at 4.8 mPa (0.048 dyn cm^−2^).

Piezo1 can directly sense mechanical force through shear stresses, as seen in Piezo1‐expressing endothelial cells.^[^ ^5^ ^]^ Piezo1 activation thresholds have been identified between 1 and 70 dyn cm^−2^ (**Table** **3**).^[^ ^4^, ^5^, ^23^ ^]^ Although there are a range of reported threshold stimulation values (Figure 2B), in most studies shear forces toward the lower end of the range (0‐2 dyn cm^−2^) do not result in Piezo1 activation, though there are a few cases where the reported threshold shear forces are substantially higher. Notably, Ranade et al. observed that shear stresses between 52 and 70 dyn cm^−2^ are required to stimulate Piezo1‐IRES‐eGFP transfected in HEK293T cells with an SS_50_ at 57 ± 3 dyn cm^−2^, with mostly no Piezo1 activation detected between 23 and 40 dyn cm^−2^.^[^ ^4^ ^]^ This decrease in sensitivity possibly stems from using different measuring systems, as the perfusion tube shear stress stimulation was combined with whole‐cell current recording, as opposed to the standard calcium imaging for shear stress experiments. Furthermore, accurately quantifying the pressure that the cell receives is more challenging in this method (Figure 2B). This could also explain the lack of Piezo1 activation observed at 18 dyn cm^−2^, when cells are stimulated with SAW.^[^ ^103^ ^]^ Moreover, most of the relatively high shear stresses that did not result in Piezo1 activation, are measured in Piezo1 transient transfected cells, compared to stable or endogenous Piezo1 expressing cells, potentially indicating that the expression system leads to different mechanosensitivity. Interestingly, different magnitudes of shear may lead to different Ca^2+^ signaling pathways, as a force of 10–12 dyn cm^−2^ caused a sustained elevation of Ca^2+^ by Piezo1 activation in HUVECs while lower forces (2‐3 dyn cm^−2^) produced a more transient increase.^[^ ^104^ ^]^ HUVECs, however, are known to natively express at least two mechanosensitive proteins, Piezo1 and TRPV4, where each may play different roles in shear sensing.^[^ ^104^ ^]^ Furthermore, studies have demonstrated that the mechanical properties of the substrate can modulate Piezo1 activity, highlighting the crucial role of the cell‐substrate interface in Piezo1 signaling.^[^ ^64^ ^]^ Given the high variability within experimental devices that are currently fabricated and utilized, a more elaborate investigation on the effect of the wide range of accurately controlled and quantified shear stresses on mechanosensitive protein activity is required.

**Table 3:** Piezo1 activation thresholds through shear forces.

Channel origin	Cell type	SS_50_	Activation shear force [dyn cm^−2^]	No activation [dyn cm^−2^]	Type of shear force	Read‐out assay and buffer	Stimulation platform	Validation method	Reference
Piezo1 (endogenous)	HAECs (human aortic endothelial cells)	N/A	6, 10, 20	0.6, 1, 2	Laminar steady	Ca^2+^ dye Fluo‐4 AM in HBSS buffer	Microfluidic Flow chamber	GsMTx4	[105]
Piezo1 (endogenous)	HUVECS	N/A	65 (6.5 Pa)		Disturbed flow (0.2s on and 2s off at 60 Hz for 40s)	Ca^2+^ dye Fluo‐3 in HBSS buffer supplemented with 10 mM HEPES	384‐Piston array	siRNA hPiezo1 depletion	[36]
Piezo1 (endogenous)	HUVECS	N/A	4 and 12		Laminar steady	Ca^2+^ dye 6‐QF in HBSS buffer supplemented with 2 mM Ca^2+^	Ibidi GmbH flow chamber	GsMTx4	^[^ ^104^ ^]^
Piezo1 (endogenous)	HUVECs	N/A	25		Laminar steady	Ca^2+^ dye Fura‐2 AM in 130 mM NaCl, 5 mM KCl, 8 mM D‐Glucose, 10 mM HEPES, 1.2 mM MgCl_2_, 1.5 mM CaCl_2_ buffer.	Ibidi GmbH flow chamber	siRNA Piezo1 depletion	[5]
hPiezo1 (endogenous)	Rat cortical neurons	N/A	Range 10–50 (1‐5 Pa)		Disturbed flow (60 Hz oscillation, 9 stimuli of 3s, 57s pause)	Ca^2+^ sensor GCaMP6S	96‐Piston array	GsMTx4	[62]
hPiezo1 (endogenous)	Rat hippocampal neurons	N/A	Range 10–50 (1‐5 Pa)		Disturbed flow (60 Hz oscillation, 9 stimuli of 3s, 57s pause)	Ca^2+^ sensor GCaMP6S	96‐Piston array	GsMTx4	[62]
Piezo1 (endogenous)	MDCK	N/A	1.1		Laminar steady	Ca^2+^ dye Fluo‐4 AM (5 µM) in an isotonic saline solution containing 1 mM CaCl_2_	Microfluidic Flow chamber	GsMTx4, Gd^3+^, Ca^2+^ free media, miRNA Piezo1, Piezo1 knockdown cells	[106]
Piezo1 (endogenous)	HPAE (endothelial)	N/A	5, 10	2	Laminar steady	Ca^2+^ dye Fura‐2 AM (3 µM) in phenol‐free media. Endoplasmic reticulum (ER) Ca^2+^ sensor G‐CEPAIA1er	Ibidi GmbH flow chamber	siRNA Piezo1 depletion/non‐transfected	[107]
Piezo1 mCherry (stable)	HEK293T	N/A	2 and 10		Laminar steady	Ca^2+^ dye Fluo‐4 AM (1 µM) in HBSS buffer supplemented with 10 mM HEPES	Ibidi GmbH flow chamber	Non‐transfected HEK293T	[29]
mPiezo1 (transfection)	HEK293T (Piezo1 Knockout)	N/A	12		Laminar steady	Ca^2+^ dye 6‐QF in HBSS buffer supplemented with 2 mM Ca^2+^	Ibidi GmbH flow chamber	Non‐transfected HEK293T‐KO‐Piezo1	[104]
mPiezo1 (transfection)	HEK293T	57 ± 3	Range of 52 ± 3–64 ± 2 (tested 0–60 mmHg equals 23.3 ± 2.2 to 70.2 ± 4.2 dyn cm^−2^)	23.3 to 40	Laminar steady	Current	Perfusion tube and recording electrode	HEK293T transfected with IRES‐eGFP	[4]
Piezo1 mCherry (stable)	HEK293T	N/A	6, 10 and 20	0.6 and 2	Laminar steady	Ca^2+^ dye Fluo‐4 AM in HBSS buffer containing 1 mM MgCl_2_, 2 mM CaCl_2_ and supplemented with 10 mM HEPES	Microfluidic Flow chamber	Non‐transfected HEK293T	[105]
mmPiezo1 (transfected)	HEK293T	N/A	N/A	34	Laminar steady	Ca^2+^ dye Fura‐2 AM in HBSS buffer supplemented with 10 mM HEPES	Ibidi GmbH flow chamber	siRNA Piezo1 depletion, non‐transfected	[36]
Piezo1 mCherry (stable)	HEK293T	N/A	20 and 60		Laminar steady	Ca^2+^ dye Fluo‐4 AM (5 µM) in HBSS buffer containing 1 mM MgCl_2_, 2 mM CaCl_2_ and supplemented with 10 mM HEPES	Ibidi GmbH flow chamber	N/A	[108]
Piezo1 (transfected)	HEK293T	N/A	10, 15 and 20	5	Laminar steady	Ca^2+^ dye Fura‐2AM in 130 mM NaCl, 5 mM KCl, 8 mM D‐Glucose, 10 mM HEPES, 1.2 mM MgCl_2_, 1.5 mM CaCl_2_	Ibidi GmbH flow chamber	siRNA Piezo1 depletion	[5]
mPiezo1 (transfected)	HEK293T‐P1KO cells	N/A	28, 50, 74	18	Acoustic streaming	Ca^2+^ dye Fura‐2AM	33‐MHz VD‐SAW transducer	HEK293T‐P1KO cells	[103]

#### 2.1.8 Acoustic Methods

Contact‐free stimulation of mechanosensitive channels through ultrasound exposure creates pressures via two main mechanisms, including acoustic radiation forces and flow induced by acoustic propagation (acoustic streaming). These forces can either act on the mechanosensitive channels themselves or indirectly through deforming the cell membrane.^[^ ^52^, ^109^, ^110^, ^111^ ^]^ Ultrasound sensitivity has been observed for Piezo1, MscL, and TRPA1.^[^ ^31^, ^69^, ^112^, ^113^, ^114^ ^]^ Chu et al. explored the use of low‐intensity ultrasound for the activation of mechanosensitive ion channels by providing a clear overview of where reported Piezo1 activation lies between 0.5 and 43 MHz.^[^ ^52^ ^]^ The standard method for characterizing mechanosensitive protein responses to ultrasound requires the use of ultrasound transducers (Figure 1I), which are often bulky, potentially obstruct the optical path for fluorescence microscopy read‐outs, and where integration with patch‐clamping technique is incompatible as low‐frequency ultrasound (≈0.2‐3 MHz) disrupts the giga‐ohm seal.^[^ ^31^, ^115^ ^]^

Recently, however, in vitro acoustic devices have explored surface acoustic waves (SAW) for small‐scale acoustic cell manipulation and characterization studies (Figure 1J). SAW is ideal for on‐chip characterization studies and is generated via patterning interdigitated electrodes on a piezoelectric substrate, and generates effects including mechanical displacement, acoustic pressures, and acoustic streaming.^[^ ^116^ ^]^ SAW exposure leads to changes in cell activity by acting on the ion channels, membrane, cytoskeleton, and extracellular matrix, thereby evoking action potentials in activating mechanosensitive channels such as Piezo1 and MscL.^[^ ^69^, ^117^, ^118^ ^]^ SAW platforms are also compatible with patch‐clamping and Ca^2+^ imaging to obtain sub‐second resolution responses.

Due to the nature of ultrasound and SAWs, however, the exact type and magnitude of the force cells encounter are challenging to accurately determine. In SAW microfluidic platforms, SAW creates acoustic streaming, which may result in flow‐induced shear stress activation, rather than direct mechanosensitive protein activation. Moreover, acoustic fields are characterized by a wide range of parameters, such as frequency, intensity, duration, and duty cycle, with subtle variations of these parameters potentially leading to inconsistent experimental outcomes.^[^ ^48^ ^]^ Another important challenge for force applied through ultrasound is to translate these parameters into units of measurement that are commonly used to quantify applied forces. Further exploration is required to elucidate the impact of these parameters on the sum of the produced mechanical force and the effect ultrasound has on mechanosensitive proteins.

#### 2.1.9 Cell Stretching Devices

Whereas the stretch applied by patch‐clamp has limited physiological relevance and is moreover only able to examine one region of a single cell at a time, cell stretching devices offer the ability to examine whole‐cell responses across large numbers of cells simultaneously. These typically utilize elastic polymer chambers, often PDMS, under different stretching modes (uni‐axial, bi‐axial or multi‐axial stretch), thereby non‐invasively applying stretch to cell populations (Figure 1K).^[^ ^119^, ^120^ ^]^ The IsoStretcher system, for example, uses PDMS stretch chambers of tuneable stiffness and studied both 2D adhered cells and 3D hydrogel‐embedded cells (Figure 1J).^[^ ^27^, ^58^ ^]^ Here, an isotropic stretch is achieved by six pins that hold a PDMS chamber that can be either stretched or relaxed by moving away or toward each other. A more updated version of the IsoStretcher applies an automated process for single‐cell quantification of a population, where they showed Piezo1 activation by stretch in adherent cardiac HL‐1 cells by Ca^2+^ imaging.^[^ ^58^ ^]^ However, the substrate relevance needs to be considered to match the complex environment in vivo.^[^ ^121^ ^]^

## 3 Measuring Intracellular and Physiological Responses

### 3.1 Electrophysiological Recording Methods

Among these techniques, patch clamp electrophysiology is considered the gold standard for measuring ion channel activity.^[^ ^122^, ^123^ ^]^ This method measures ion flux across the membrane and the gating mechanisms of even single ion channels, usually via changes in the membrane potential or by adding channel agonists. During patch clamping, a high‐resistance giga‐ohm seal is formed between the cell membrane and a glass pipette containing an internal solution and a recording electrode. In the current clamp mode, a current is applied by the patch pipette, and the membrane potential is recorded, allowing detection of action potentials when the ion channel is stimulated. This is typically used to monitor resting membrane potential and measure action potentials in electrically excitable cells such as neurons. In the case of a voltage clamp, a single membrane potential is kept at a set value to detect the membrane current of individual ion currents to study specific ion channels. This is a desirable method for recording current movement through voltage‐gated or ligand‐gated ion channels. Alternatively, a non‐invasive Multielectrode arrays (MEA) system, consisting of dot‐like electrode structures arranged in a 2D grid, measures the extracellular field potential of a population of cells, allowing long‐term and high throughput recording.^[^ ^124^ ^]^ Such electrophysiological recording methods are complementary with various techniques of mechanical stimulation that apply mechanical forces to probe mechanosensitive ion channels, including pressure clamps, AFM, micropillars, ultrasound transducers, and stretching devices.

### 3.2 Optical Techniques

Electrophysiological methods obtain data on the gating mechanisms of mechanosensitive channels via measurement of membrane currents. One fundamental limitation of these techniques, e.g. patch clamps and MEA, is the difficulty in obtaining information about specific molecular response mechanisms within the cell itself in response to mechanical stimulation. In addition, most such techniques have limited throughput, are invasive/damaging to the cell, are obtained in setups not necessarily reflective of physiological conditions and require special technical expertise and instrumentation. Investigating the underlying cellular mechanisms by optical techniques, however, including downstream signaling pathways and their mechanism of action, provides a less invasive and higher throughput approach suitable for long‐term intracellular recording. Visualizing the signaling pathways is a powerful tool to measure the real‐time dynamic responses of mechanosensitive proteins. In addition, fluorescent tools provide readouts on membrane tension without physically perturbing the membrane compared to micropipette aspiration and AFM. Depending on the technique, assays can monitor protein activity proximal to the mechanosensitive ion channel, reflecting the direct influences of mechanotransduction on cell homeostasis. Some notable approaches are described here.

#### 3.2.1 Direct Recording of Mechanosensitive Activation

**Figure 3:**
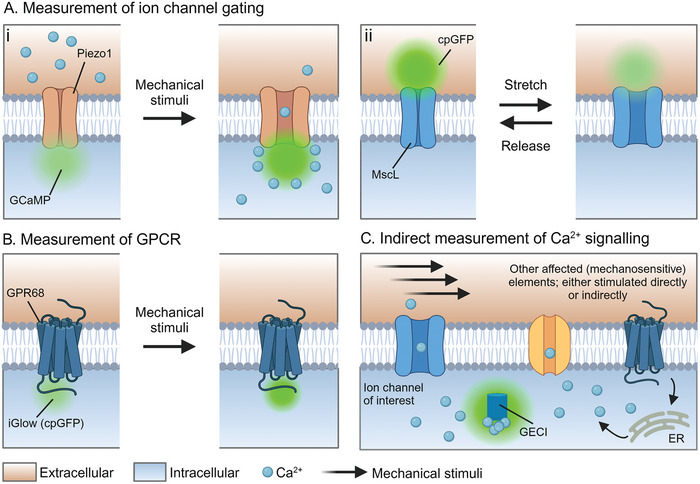
Optical approaches to studying mechanosensitive protein responses. A) Membrane tension approaches for direct measurement of mechanosensitive ion channels, such as i) MscL^[^ ^130^ ^]^ and ii) Piezo1,^[^ ^125^ ^]^ upon mechanical stimulation. B) Fluorescent receptor approach for direct measurement of mechanosensitive GPCR, for example, GPR68.^[^ ^126^ ^]^ C) When mechanical stimulation induces channel opening, Ca^2+^ accumulation leads to Ca^2+^ binding to intracellular located GECI, causing an increase in fluorescence. Potentially, other mechanosensitive elements, directly or indirectly stimulated by mechanosensitive stimulation, can contribute to Ca^2+^ accumulation, thereby causing GECI fluorescence increase. Created with Biorender.

Optical recording of cellular processes upon mechanosensitive protein activation is valuable for the identification and visualization of signaling pathways, both intracellularly and within membranes. The development of molecular assays that reflect the direct involvement of the mechanosensitive protein of interest offers insights into the precise activation mechanism, where Ca^2+^ signaling can readily be used to assess protein activation (**Figure** **3**Ai). For example, Yaganoglu et al. engineered GenEPI, a Piezo1‐specific mechanosensitive reporter by targeting a genetically encoded Ca^2+^ indicator (GECI) to the C‐terminus of Piezo1, which resides intracellularly.^[^ ^125^ ^]^ A GenEPi‐expressing zebrafish transgenic line was subsequently developed to visualize Piezo1‐specific activity in vivo, providing a useful system for the testing and identification of new Piezo1 modulators that target channel function. Similarly, by inserting cyclic permuted GFP (cpGFP) into the third intracellular loop of GPR68, a region that undergoes a large conformational change upon mechanical stimulation, a GPR68‐specific fluorescence reporter named iGLow was created (Figure 3B). Here, iGlow increases in fluorescence when GPR68 becomes activated by shear stress and acidification.^[^ ^126^ ^]^ However, the sensor showed unspecific responses and large cell‐to‐cell fluctuations, highlighting the complexity in creating and utilizing a mechanosensitive protein‐specific reporter.

The development of optical membrane tension sensors, which repurposes mechanosensitive proteins that respond to membrane tension changes, further represents a valuable tool for measuring membrane tension and associated mechanosensitive protein responses.^[^ ^127^, ^128^, ^129^ ^]^ Compared to micropipette aspiration and AFM, membrane tension sensors provide readouts on membrane tension without physically perturbing the membrane and are especially beneficial in measuring mechanotransduction responses in vivo where other quantification methods such as micropipette aspiration are not possible. For instance, Hsu et al. engineered a tension sensor by inserting a GFP mutant into the mechanosensitive channel MscL (Figure 3Aii). An increase in relative membrane tension induces a conformational shift in the transmembrane helices of this engineered sensor, resulting in channel opening and switching the GFP mutant from fluorescent (low tension) to low fluorescence (high tension).^[^ ^130^ ^]^

Another approach to monitor real‐time, mechanosensitive protein activation or mechanotransduction activities is by fluorescence (FRET) or bioluminescence (BRET) resonance energy transfer assays.^[^ ^131^, ^132^ ^]^ These techniques rely on the transfer of energy from a donor fluorophore (or luminescent protein) to an acceptor fluorophore when near each other. The energy transfer may be disrupted or augmented by force‐induced deformations in the protein, whereby the FRET/BRET signal is reflective of the applied stress. The major difference between the two is that FRET requires excitation by a light source to excite the fluorophore, whereas BRET requires a substrate for a luminescent RET donor and is independent of external light excitation. Both techniques have been widely used for monitoring ligand binding and cell signaling in the GPCR research field and have also been implemented for mechanical stimuli sensing and ion channel triggering.^[^ ^133^, ^134^, ^135^, ^136^ ^]^ Interestingly, applying force transmission sensors to elements of the cytoskeleton and ECM, such as the F‐actin architecture, reflects the internal distribution of forces upon mechanical stimuli in a direct observational manner, a crucial element not captured by traditional Ca^2+−^based assays.^[^ ^134^, ^137^ ^]^ However, more research on FRET/BRET sensors is still required to overcome the challenges associated with labeling residues within an ion channel protein with fluorophores, such as affecting the channel gating, which still hinders the broader applicability of these reporters for mechanotransduction assays.^[^ ^138^ ^]^

#### 3.2.2 Indirect Recording of Mechanosensitive Activation

The activation of mechanosensitive proteins typically generates a series of downstream cellular signalling pathways that in turn regulate biochemical responses and gene expression.^[^ ^49^, ^74^, ^139^, ^140^, ^141^ ^]^ Therefore, linking the biochemical signals to force‐induced signals reflects molecular mechanisms underlying the mechanosensitive protein response. Ca^2+^ ions are a ubiquitous second messenger for many downstream signalling pathways and lead to the activation of several kinases and phosphatases, muscle contraction, and neurotransmitter release.^[^ ^142^, ^143^ ^]^ Measuring cytoplasmic Ca^2+^ concentrations in response to mechanosensitive protein activation is common, often using Ca^2+^ indicator dyes such as Fura‐2 and Fluo‐4. These dyes can cross cell membranes and bind to free cytoplasmic Ca^2+^ ions, resulting in a 50‐fold increase in fluorescence.^[^ ^144^ ^]^ Notably, Ca^2+^ dyes have been found to negatively impact a number of cellular functions, including the sodium‐potassium pump Na,K‐ATPase.^[^ ^145^, ^146^ ^]^ Alternatively, genetically encoded Ca^2+^ indicator (GECI) proteins, such as GCaMP and R‐GECO, can be used to measure Ca^2+^ influx through the induction of a conformational change that results in increased fluorescent emission (Figure 3C).^[^ ^38^, ^147^ ^]^ GECIs can be targeted to specific cell compartments or genetically expressed in specific cell types, allowing for a wide variety of experimental applications in both in vitro and in vivo imaging.^[^ ^148^ ^]^ GECIs offer improved dispersion in the cytosol of cells, and no leakage of dye out of the cell compared to fluorescent dyes.^[^ ^149^ ^]^ However, GECIs usually have slow on‐and‐off Ca^2+^ binding rates, limiting the temporal resolution of rapid Ca^2+^ concentration changes.

Similarly, mechano‐activation of mechanosensitive GPCRs, such as angiotensin II type 1 receptor (AT1R) and G protein‐coupled receptor 68 (GPR68) leads to diverse downstream signaling events, most notably leading to activation of G‐proteins.^[^ ^39^, ^150^ ^]^ High throughput assays such as CRE/β‐galactosidase‐based colorimetric assays that detect intracellular accumulation of cAMP or Ca^2+^, provide insight into the specific activation of Gs‐ and Gq‐coupled receptors.^[^ ^151^ ^]^ However, it is important to consider that indirectly measuring channel activity by monitoring downstream in the signaling pathway can result in false positives, as Ca^2+^ increases may arise from other targets in the pathway, and thus are a less specific indication of mechanosensitive ion channel or GPCR response requiring carefully controlled experiments.^[^ ^18^ ^]^ Nevertheless, molecular assays that reflect the expressed pathway of a single mechanosensitive protein have the potential to allow for accurate and reliable quantification of both mechanosensitive protein activity while elucidating signalling pathway dynamics. Furthermore, specific inhibitors for mechanosensitive ion channels and GPCRs are important pharmacological tools for determining the contribution to the response, however such antagonists are limited. Currently, peptide GsMTx4 is the only known inhibitor to target specific cationic mechanosensitive channels, including Piezo1 and TRP ion family, however, is therefore non‐specific to Piezo1.^[^ ^152^, ^153^, ^154^ ^]^ Notably, GsMTx4 does not directly block the ion channel pore, but rather distorts the membrane tension near the channel, thereby reducing the force magnitude acting on mechanosensitive ion channels. Therefore, small‐molecule inhibitors specific to individual mechanosensitive proteins are largely lacking and a challenge in the field.

### 3.3 Reconstitution of Mechanosensitive Proteins in Artificial Bilayers

A common approach to studying mechanosensitive proteins involves overexpressing exogenous mechanosensitive proteins, thereby deliberately making cells hypersensitive to mechanical stimuli. Consequently, this results in increased expression levels of mechanosensitive channels, thus increasing the signal obtained during mechanical stimulation. However, a common hurdle to studying mechanosensitive ion channels is the inevitable activation of non‐target, ubiquitously expressed mechanosensitive proteins. Even in the absence of endogenous mechanosensitive proteins, cytoskeletal elements can still tune the sensitivity and thereby influence mechanosensitive channel behavior.^[^ ^29^, ^70^ ^]^ Accordingly, an acellular approach can be useful for reproducible quantification, wherein reconstituted purified ion channels are inserted into artificial lipid bilayers. This controlled approach allows for the investigation of mechanosensitive channel gating requirements and the stoichiometry needed for protein response.^[^ ^155^ ^]^

Artificial lipid bilayers can be generated in a diverse range of formats and techniques, including planar lipid bilayers (i.e., black lipid membranes, solid‐supported lipid bilayers, and tethered bilayer lipid membranes) and unilamellar versicles in diverse sizes, from small (r<100 nm) to giant (r>1 µm) vesicles.^[^ ^156^, ^157^ ^]^ Over the years, several mechanosensitive ion channels, including MscL^[^ ^21^, ^158^ ^]^ MscS,^[^ ^159^ ^]^ TRPA1,^[^ ^12^ ^]^ Piezo1,^[^ ^14^, ^32^ ^]^ TRAAK, and TREK1,^[^ ^160^ ^]^ have been successfully reconstituted into artificial bilayers and activated by mechanical forces including membrane stretch, membrane indentation, and ultrasound. In 1995, purified MscL was found to be fully functional after reconstitution into liposomes.^[^ ^161^ ^]^ Moreover, Piezo1 reconstitution led to the discovery that Piezo1 is inherently mechanosensitive, where membrane properties play a crucial role in Piezo1 gating.^[^ ^32^, ^33^ ^]^ Interestingly, Rosholm et al. measured the threshold membrane tension in droplets on hydrogel bilayers (DHBs) required to activate the MscL channel as a function of injected volume.^[^ ^162^ ^]^ Elevating the volume to create a systematically asymmetric bilayer tension resulted in the activation of MscL channel, where the tension at which half of the channels were open (T_50_) was measured at 9 ± 1 mN m^−1^. Due to their similarity in size and curvature to mammalian eukaryotic cells, giant unilamellar vesicles (GUVs) are also a promising and well‐suited in vitro system for the incorporation of mechanosensitive ion channels. GUVs have been shown to support the function of transmembrane proteins, allowing real‐time monitoring of mechanosensitive protein behaviour by standard optical microscopy techniques.^[^ ^158^, ^163^, ^164^, ^165^, ^166^ ^]^ Furthermore, micropipette aspiration can be applied to both liposomes and GUV systems to control membrane tension, offering a valuable tool for studying channel activity and channel/membrane interactions.^[^ ^155^, ^163^, ^167^ ^]^ The control of the mechanical stimuli and the specific mechanosensitive channel without any external variables makes this platform well‐suited for investigating controlled force versus response measurements.

In spite of these advantages, ion channel reconstitution into lipid bilayers remains a challenging procedure with difficulties in obtaining a high level of protein expression for subsequent purification, reconstitution in the required amounts, and preserving functionality and orientation.^[^ ^168^, ^169^ ^]^ In addition, mechanosensitive protein reconstitution investigation is largely limited to membrane channels given that the absence of endogenous systems complicates the study of signaling cascades induced by channel activation as seen in GPCRs,^[^ ^170^ ^]^ though efforts are being made toward building cell‐like systems with reconstituted cell functionalities, including a synthetic route for biomimicry of a mechanosensitive signaling pathway.^[^ ^171^, ^172^ ^]^ Given that the cell environment and membrane constituents strongly influence the kinetics of channels, incorporating this is especially interesting for assessing how membrane properties influence mechanosensitive protein gating and sensitivity.

## 4 Physiological Models

Mechanotransduction is crucial for maintaining various cellular functions, and modifications in mechanosensitive protein organization are linked to diseases, including hearing loss and cancer.^[^ ^173^, ^174^ ^]^ Mechanotransduction disorders can result from alterations in cytoskeletal structures, mechanical properties such as membrane tension, or downstream signaling pathways. While many studies focus on cell culture or reconstituted systems, in vivo models are essential for understanding physiologically relevant mechanisms and diseases. For example, when comparing in vivo versus in vitro endothelial gene expression levels, it was found that many in vivo mechanosensitive genes were either dysregulated or lost, due to phenotypic changes in cultured cells.^[^ ^175^ ^]^ This highlights that, while in vitro stimulation is valid and provides useful insight into mechanosensitive proteins, in vivo models ensure accurate insights into physiologically relevant mechanisms, roles in disease and offer valuable information for translating mechanosensitive stimulation for therapeutic purposes.^[^ ^41^, ^76^, ^77^, ^78^, ^79^, ^80^, ^81^, ^82^, ^83^, ^84^, ^85^, ^86^, ^87^, ^88^, ^89^, ^90^, ^91^, ^92^, ^93^, ^94^, ^95^, ^96^, ^97^, ^98^, ^99^, ^100^, ^101^, ^102^, ^103^, ^104^, ^105^, ^106^, ^107^, ^108^, ^109^, ^110^, ^111^, ^112^, ^113^, ^114^, ^115^, ^116^, ^117^, ^118^, ^119^, ^120^, ^121^, ^122^, ^123^, ^124^, ^125^, ^126^, ^127^, ^128^, ^129^, ^130^, ^131^, ^132^, ^133^, ^134^, ^135^, ^136^, ^137^, ^138^, ^139^, ^140^, ^141^, ^142^, ^143^, ^144^, ^145^, ^146^, ^147^, ^148^, ^149^, ^150^, ^151^, ^152^, ^153^, ^154^, ^155^, ^156^, ^157^, ^158^, ^159^, ^160^, ^161^, ^162^, ^163^, ^164^, ^165^, ^166^, ^167^, ^168^, ^169^, ^170^, ^171^, ^172^, ^173^, ^174^, ^175^, ^176^, ^177^, ^178^ ^]^ Additionally, organoids hold promise for more physiologically relevant and personalized in vitro experimentation tools. Organoid cultures recreate in vivo‐like interactions by growing cells in a 3D tissue environment whose structure may resemble that of native tissues on a physical and functional basis, representing an attractive platform for disease modeling.^[^ ^179^, ^180^, ^181^ ^]^ Therefore, assembling organoids in microfluidic platforms represents an effective approach to recapitulating realistic in vitro models, with various complexities and biologically relevant structures to study mechanotransduction signaling pathways more analogous to in vivo contexts.^[^ ^182^ ^]^

Quantitative in vivo cellular recording methods, such as MEAs or recording bioelectric potential differences in muscle tissue using electromyography (EMG) allow for the investigation of the excitatory and inhibitory effects upon mechanical stimulation.^[^ ^66^, ^183^ ^]^ While these techniques do not provide direct data on specific protein channel gating responses, they do provide necessary evidence about the interplay between the activation of mechanosensitive proteins upon stimulation and resultant electrophysiological effects.

Emerging technologies for in vivo investigation are based on either implanted devices, such as electrodes or optogenetics approaches, or external tools using ultrasound. As ultrasound can transmit through intact thin bone and deep tissue, this approach allows for therapeutic applications by non‐invasively modulating neural activity.^[^ ^113^, ^184^, ^185^ ^]^ Modifying cellular mechano‐sensitivity accordingly offers a versatile approach for targeted neuronal stimulation. Sonogenetics, which involves engineering cells to express mechanosensitive channel proteins, enables the modulation and control of acoustic‐sensitive neurons.^[^ ^11^ ^]^ Low‐intensity ultrasound (<3.5 MHz) has been mainly utilized for neuromodulation because it provides high spatiotemporal precisions as well as deep soft‐tissue penetration, through nonthermal effects.^[^ ^115^ ^]^ For example, the overexpression of Piezo1 in HEK293T cells enables cells to respond to low‐intensity, low‐frequency ultrasound.^[^ ^30^ ^]^ Achieving cell‐specific selectivity, however, remains challenging due to the diverse cell types and mechanosensitive proteins in brain tissue. A deeper understanding of the properties of stimuli required to activate various channels is crucial for accurately activating and recording mechanosensitive proteins in vivo, ensuring optimal responses while minimizing off‐target effects, for example by combining accurate stimuli (e.g., implanted ultrasound transducers/SAW device) close to the therapeutic target that is tagged with a specific fluorescent reporter (e.g., GECI specific to ion channel studied).^[^ ^186^, ^187^ ^]^ Ultimately, target‐specific effects over background could be defined by incorporating a specific small molecule or antibody inhibitor.

## 5 Conclusion and Outlook

Techniques to study cell mechanics have aided the investigation of how cells sense their mechanical environment, though there is no single ideal approach to quantify the force versus response of mechanosensitive protein function. Mechanical stimulus via indentation, fluid shear stress, ultrasound, and elastomeric pillar deflection have all been used to measure activation of Piezo1, for example, but a well‐established quantitative relationship between applied force, the exact received force on the protein and resulting protein response remains to be determined and corroborated across different measurement modalities. In addition, many current tools only allow the measurement of a single force at a time, instead of parallel experimentation, limiting throughput and reproducibility.

Microfluidic devices are, accordingly, finding increasing use as an approach for mechanical stimulation given their physiological relevance and ability to accurately control applied shear forces. In contrast to 2D cell cultures, microfluidic approaches provide controlled and physiologically relevant conditions by incorporating complex microenvironments that play crucial roles in mechanosensing, such as tuning tissue stiffness, adding extracellular matrix substrates, and providing adjacent tissue interactions.^[^ ^188^, ^189^ ^]^ Microfluidic platforms have been combined with a variety of mechanical stimuli, including micropillar array substrates,^[^ ^190^ ^]^ AFM^[^ ^191^ ^]^ techniques, compression,^[^ ^192^ ^]^ shear stress,^[^ ^193^ ^]^ and acoustics,^[^ ^194^, ^195^ ^]^ either applying a single mechanical force or a combination of forces.^[^ ^196^ ^]^ Furthermore, microfluidic platforms can aid the physical trapping of individual cells or GUVs for imaging, media exchange, and labeling, while simultaneously being exposed to a mechanical stimulus.^[^ ^197^, ^198^, ^199^, ^200^ ^]^ Additionally, the non‐contact arrangement of cells in fluid can be achieved through the patterning of cells with SAW, facilitating the investigations of single cells.^[^ ^201^, ^202^, ^203^ ^]^

Mechanosensation is a vital, yet poorly understood process in biology. Identifying the exact forces a mechanosensitive protein experiences upon stimulation remains a challenging endeavour, where ongoing research and technical developments in computational and mechanotyping techniques can advance our understanding.^[^ ^204^ ^]^

## Conflict of Interest

The authors declare no conflict of interest.
